# Trajectories of care of community-dwelling people living with dementia: a multidimensional state sequence analysis

**DOI:** 10.1186/s12877-023-03926-x

**Published:** 2023-04-27

**Authors:** Isabelle Dufour, Isabelle Vedel, Josiane Courteau, Amélie Quesnel-Vallée

**Affiliations:** 1 Department of Epidemiology, Biostatistics, and Occupational Health, Faculty of Medicine, McGill University, 2001 McGill College, Suite 1200, Montreal, Qc H3A 1G1 Canada; 2 Department of Family Medicine, Faculty of Medicine and Health Sciences, Faculty of Medicine, McGill University, 5858 Chemin de La Côte-Des-Neiges, Montreal, Qc H3S 1Z1 Canada; 3 Groupe de Recherche PRIMUS, Centre de Recherche du Centre Hospitalier Universitaire de Sherbrooke (CRCHUS), 12e Avenue N, Sherbrooke, QC J1H 5N4 Canada; 4 Department of Sociology, Faculty of Arts, McGill University, 855 Sherbrooke Street West, Montreal, Qc H3A 2T7 Canada

**Keywords:** Dementia, Alzheimer’s disease, Care trajectory, State sequence analysis, Healthcare services

## Abstract

**Background:**

The type and level of healthcare services required to address the needs of persons living with dementia fluctuate over disease progression. Thus, their trajectories of care (the sequence of healthcare use over time) may vary significantly. We aimed to (1) propose a typology of trajectories of care among community-dwelling people living with dementia; (2) describe and compare their characteristics according to their respective trajectories; and (3) evaluate the association between trajectories membership, socioeconomic factors, and self-perceived health.

**Methods:**

This is an observational study using the data of the innovative Care Trajectories -Enriched Data (TorSaDE) cohort, a linkage between five waves of the Canadian Community Health Survey (CCHS), and health administrative data from the Quebec provincial health-insurance board. We analyzed data from 690 community-dwelling persons living with dementia who participated in at least one cycle of the CCHS (the date of the last CCHS completion is the index date). Trajectories of care were defined as sequences of healthcare use in the two years preceding the index date, using the following information: 1) Type of care units consulted (Hospitalization, Emergency department, Outpatient clinic, Primary care clinic); 2) Type of healthcare care professionals consulted (Geriatrician/psychiatrist/neurologist, Other specialists, Family physician).

**Results:**

Three distinct types of trajectories describe healthcare use in persons with dementia: 1) low healthcare use (*n* = 377; 54.6%); 2) high primary care use (*n* = 154; 22.3%); 3) high overall healthcare use (*n* = 159; 23.0%). Group 3 membership was associated with living in urban areas, a poorer perceived health status and higher comorbidity.

**Conclusion:**

Further understanding how subgroups of patients use healthcare services over time could help highlight fragility areas in the allocation of care resources and implement best practices, especially in the context of resource shortage.

**Supplementary Information:**

The online version contains supplementary material available at 10.1186/s12877-023-03926-x.

## Background

Dementia is considered a primary health concern, as individuals living with such conditions often experience severe and persistent disabilities, participation restrictions, and high formal and informal care needs. The World Health Organisation recognizes dementia as a public health priority [1]. Accordingly, many North American jurisdictions have adopted large-scale policies oriented towards improving health equity, access to healthcare, and quality of life for people living with dementia and their care partners [2, 3].

Yet, despite significant improvements in recent years, various barriers to optimal care remain. Indeed lack of care coordination, suboptimal transition processes, and poor quality of care are still reported [2, 4], which may lead to adverse outcomes, such as higher rates of avoidable hospitalization and emergency departments (ED) visits, as well as institutionalization [5, 6]. Indeed, people living with dementia experience diverse patterns of healthcare use.

Dementia is characterized by three stages (early, middle, and advanced stages), where the type and level of services required to address the needs of individuals may fluctuate significantly [7]. Throughout disease progression, these needs are further complexified by higher rates of multimorbidity and polypharmacy [8, 9]. Moreover, socioeconomic determinants further exacerbate these difficulties in accessing and navigating the healthcare system, as socioeconomic deprivation is associated with more ED visits and urgent healthcare needs and negatively impacts long-term survival [10].

Thus, service improvement requires a better understanding of the complex dynamics underlying care use in populations living with dementia. This could be gained by describing trajectories of care (ToCs), i.e., the sequence of healthcare use over time [11].

Some studies have advanced our knowledge of ToCs in people living with dementia [12]. For example, a study conducted in the United States by Rahman et al. [13] aimed to describe survival and ToCs among individuals from rural and urban areas. Their results suggest that, after diagnosis, individuals from rural areas spend more time in nursing homes and receive less home health care than their urban counterparts [13]. In Canada, Bronskill et al. [14] aimed to determine the long-term ToCs by a general group of individuals with dementia as they remain in the community over time. Amongst others, they observed higher rates of health service use, long-term care placement, and mortality among individuals living with dementia compared with individuals without the condition [14]. Building on these previous studies and to bridge remaining knowledge gaps, we sought to examine multidimensional ToCs (according to the healthcare settings and the professionals consulted) while accounting for population heterogeneity [12].

We used a state sequence analysis (SSA) approach and data from the Care Trajectories—Enriched Data (TorSaDE) cohort, a novel health administrative and survey data linkage. SSA is an innovative statistical method that enables the visualization of complex longitudinal patterns [15]. Its use in healthcare research has recently increased, and to our knowledge, this is the first study to apply SSA to examine ToC in people living with dementia [12]. We operationalized the “6W” multidimensional model of ToCs in six dimensions: patients, with their individual and clinical attributes (*who*), in responding to their illness condition and care needs (*why*) will seek healthcare services from different categories of care providers (*which*) in care units and settings (*where*) where they will receive tests and treatments (*what*) at specific times (*when*) [11].

Specifically, we aimed to (1) propose a multidimensional typology of ToCs among community-dwelling people living with dementia; (2) describe and compare the characteristics of people living with dementia according to their respective ToC; and (3) evaluate the association between ToC membership, socioeconomic factors, and self-perceived health.

## Methods

### Design and data sources

This is a retrospective cohort study in the province of Quebec, Canada. The study was approved by the Commission d’accès à l’information, l’Institut de statistique du Québec, and McGill’s Research Ethics Board.

We gathered study data from the Care Trajectories—Enriched Data (TorSaDE) cohort (*n* = 102,148 distinct participants and available from 1996 to 2016), a linkage of data from two complementary sources: the Canadian Community Health Survey (CCHS) and health administrative data from the provincial health insurance boards (Régie de l’assurance maladie du Québec: RAMQ). This novel data linkage overcomes the perennial challenge that health administrative data tend to lack individual-level socioeconomic characteristics and patient-reported outcomes, such as self-rated health. The full description of the cohort is available elsewhere [16]. Briefly, the CCHS is a cross-sectional survey providing in-depth self-reported information on health status, healthcare utilization, health indicators, and sociodemographic characteristics. Its sampling base represents about 97% of the Canadian population aged 12 years or older. The TorSaDE cohort includes all Quebec (Canada) respondents who participated in at least one of the five cycles (2007–08, 2009–10, 2011–12, 2013–14, 2015–16) of the CCHS and agreed to their data to be shared (sharing consent rate of about 96% for all CCHS cycles).

In Quebec, RAMQ provides universal health insurance coverage to all residents. Coverage includes services provided in ED, hospitals, and medical clinics, including primary health care clinics. The RAMQ administrative health register gives access to an extensive range of variables, including (1) patient demographic information (date of birth, date of death if applicable, postal code); (2) medical services register (data on medical services provided by Quebec fee-for-service physicians, including diagnoses coded according to the International Classification of Diseases 9 (ICD-9)); (3) pharmaceutical services (data on pharmacy-claimed drugs, for patients covered by the public drug insurance plan – about 85% of people 65 years old and over); (4) MED-ECHO registry (information on hospitalization, including diagnosis coded in ICD-10); (5) I-CLSC database (available since April 2012 and containing information about care services provided in local community service centers (CLSC)).

### Studied population

The cohort was composed of all community-dwelling Quebec citizens who: (1) participated in at least one cycle of the CCHS — index date — (if an individual participated in more than one cycle, we kept the most recent); (2) were 65 years or older at the time of the CCHS completion; 3) had a dementia condition at the time of the CCHS completion. Patients permanently institutionalized in long-term care settings are excluded from the CCHS coverage and were thus not included in our study [16].

We used the RAMQ data to identify dementia cases using a validated algorithm (sensitivity of 79.3%, specificity of 99.1%) [17]. The definition of dementia used to develop the algorithm includes Alzheimer’s disease, vascular dementia, dementia in other diseases classified elsewhere (frontotemporal dementia, idiopathic normal pressure hydrocephalus), and unspecified dementia (senile dementia, presenile dementia). The ICD-9 and 10 codes used are: ICD-9 (46.1, 290.0, 290.1, 290.2, 290.3, 290.4, 294.x, 331.0, 331.1, 331.5, 331.82); ICD-10 (F00.x, F01.x, F02.x, F03.x, G30.x). Individuals were considered as having dementia if they met a least one of the following criteria: (1) the presence of a primary or secondary diagnosis code for dementia during hospitalization (in MED-ECHO); (2) the presence of three dementia diagnosis codes registered at least 30 days apart in two years (in the medical services register); (3) a prescription claimed for an Alzheimer's disease-related drug in the pharmaceutical services register (cholinesterase inhibitor subclass, including donepezil, galantamine, rivastigmine, tacrine, and memantine). The date of dementia identification was when the first criteria became positive.

### Variables

#### Trajectories of care

ToCs of our study population (Dimension *who*) were defined as sequences of healthcare utilization in time (dimension *when*) in the two years before the index date (date of the CCHS completion). Our ToCs were defined using the following information: 1) Dimension *where* — Care units consulted (1. Hospital, 2. ED, 3. Outpatient clinic, 4. Primary care clinic); 2) Dimension *which* — Healthcare care professionals consulted (1. Geriatrician/psychiatrist/neurologist, 2. Other specialists, 3. General practitioner). As the I-CLSC database was not available for our whole study period, we could not consider data relative to services provided in the community by nurses and other professionals in the ToCs development.

#### Demographics and clinical characteristics (dimension Who)

##### Variables identified in health administrative data

Age sex, residential neighborhood characteristics (metropolitan area: ≥ 100,000 inhabitants; small town: 10,000–100,000 inhabitants; rural: < 10,000 inhabitants) were considered at the index date. Second, health conditions were identified in the two years preceding the index date, based on two criteria: 1) two diagnosis codes for the same condition entered in the medical services register on two different dates or (2) one primary or secondary diagnosis code for the condition during hospitalization (MED-ECHO), if applicable (See Additional file 1). The comorbidity index selected is the Combined Comorbidity Index of Charlson and Elixhauser [18].

We calculated the monthly average number of specific claimed drugs two years before the index date. We computed if the individuals claimed a prescription (yes/no) of potentially inappropriate medications (benzodiazepine, anticholinergic, antipsychotic, and opioid) to bring complementary information. We estimated the maximum proportion of healthcare visits to the same provider in the two years preceding the index date to inform on care continuity in primary care settings.

##### Variables identified in the CCHS

The following variables were identified at the index date and categorized as follows: household size (living alone or not), individual annual income (≤ 20,000$, > 20,000$), education level (≤ secondary, > post-secondary), perceived health status (poor/fair, good/very good/excellent), and having a regular physician (yes/no).

### Statistical analysis

Using SSA terminology, a ToC corresponds to a sequence during which successive ‘states’ (value taken by a variable at a specific time) are encountered. The general principle of SSA is to compare sequences (i.e., individual ToCs of patients) with respect to the order in which their component states appear [19].

To propose a typology of ToCs of people living with dementia and to suit our multidimensional approach, we followed the six steps of a modified SSA approach [15], as proposed in the work of Vanasse et al. [20].

In step one, we developed ToC for people living with dementia two years before the CCHS completion (index date) and chose ‘months’ as the time unit. In step two, for each month over the two-year observation period and for all patients, we defined the dimension-specific states in priority order. Dimension *where*: 1) Hospital; 2) ED; 3) Outpatient clinic; 4) Primary care clinic; 5) No such healthcare services. Dimension *which*: 1) Geriatrician/psychiatrist/neurologist; 2) Other specialists; 3) General practitioner: 4) No such healthcare services. According to the dimension-specific states, and for each patient, we created a ToC sequence consisting of 24 states (for the 24 months preceding the index date). In step three and four, we used the dynamic hamming distance (DHD) alignment algorithm in TraMineR to derive a pooled distance matrix. [21] In step five, we used a hierarchical cluster analysis on the matrix with Ward’s criterion to create homogeneous ToC types. The optimal number of ToC types was based on statistical criteria (dendrogram and inertia curve), parsimony, interpretability, and clinical judgment [22]. In step six, we used chronograms (also referred to as state distribution plots) to show a general distribution pattern of states at each time unit and a representation of the mean duration in each state.

We described the individual’s characteristics by ToC types. The differences between subgroups were tested using ANOVA (for continuous variables) and chi-square tests (for categorical variables), with a significance level of 0.05.

Finally, we used a multinomial logistic regression model to test the association between group membership (ToC type), socioeconomic factors, and perceived health while accounting for comorbidity. The final model report odds ratios with the associated 95% confidence intervals. The two × two matrices and the variance inflation factor showed no multicollinearity issues.

SSA was performed using the TraMineR package in R [23]. All other analyses were performed using SAS Enterprise Guide 8.3 (SAS Institute, Cary, NC).

## Results

The study cohort included 690 community-dwelling individuals living with dementia. Our SSA approach revealed three groups, or ToC types, composed of individuals characterized by (1) *low healthcare use* (*n* = 377; 54.6%); (2) *high primary care use* (*n* = 154; 22.3%); and (3) *high overall healthcare use* (*n* = 159; 23.0%).

**Fig. 1:**
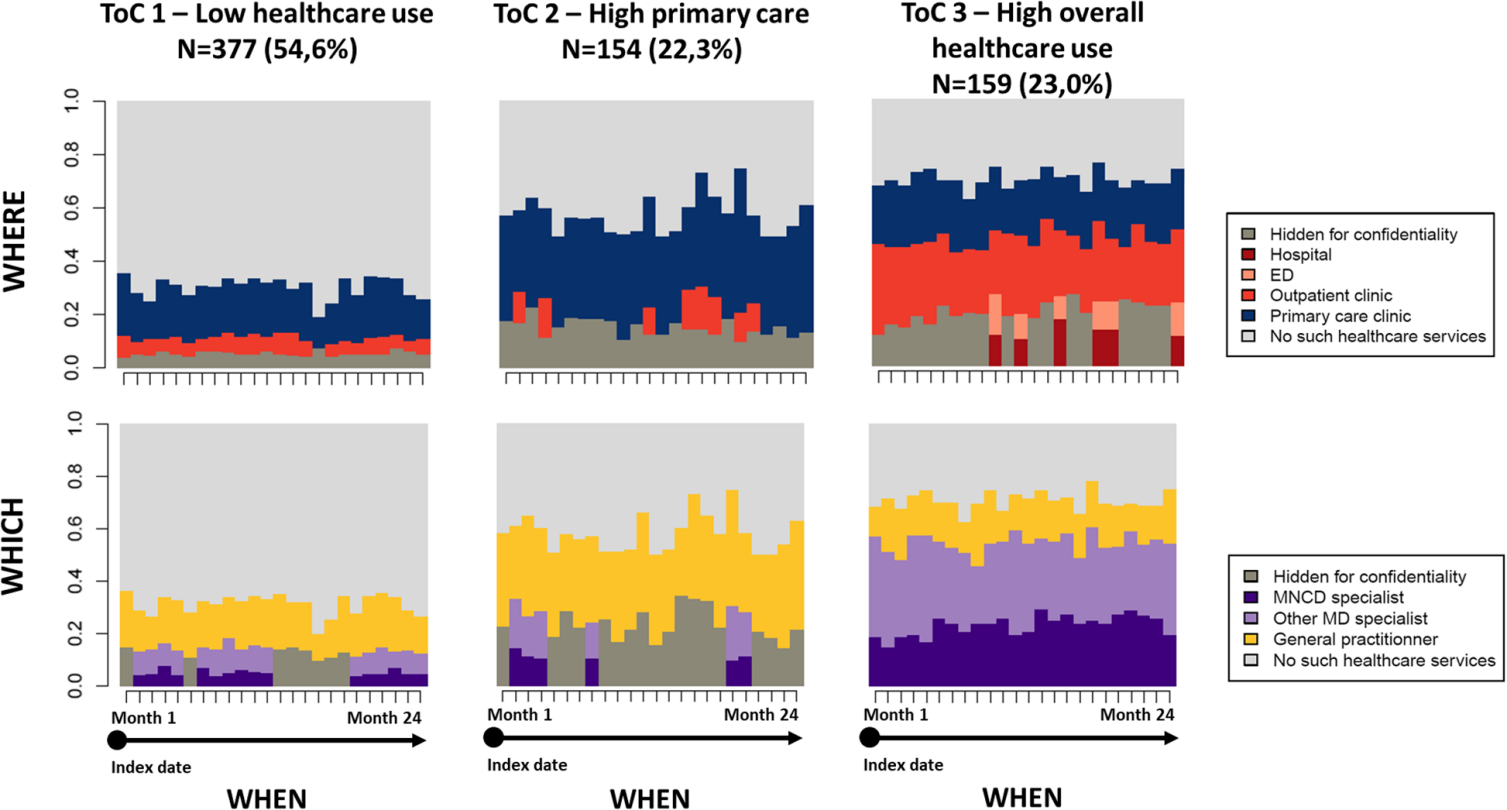
State distribution plot of ToCs of persons living with dementia, by dimension. The category ‘hidden for confidentiality’ groups together the categories not meeting the confidentiality data standards, due to an insufficient number of observations. Abbreviations: ToC, Trajectory of care; ED, Emergency department; MD, Doctor of Medicine

**Fig. 2:**
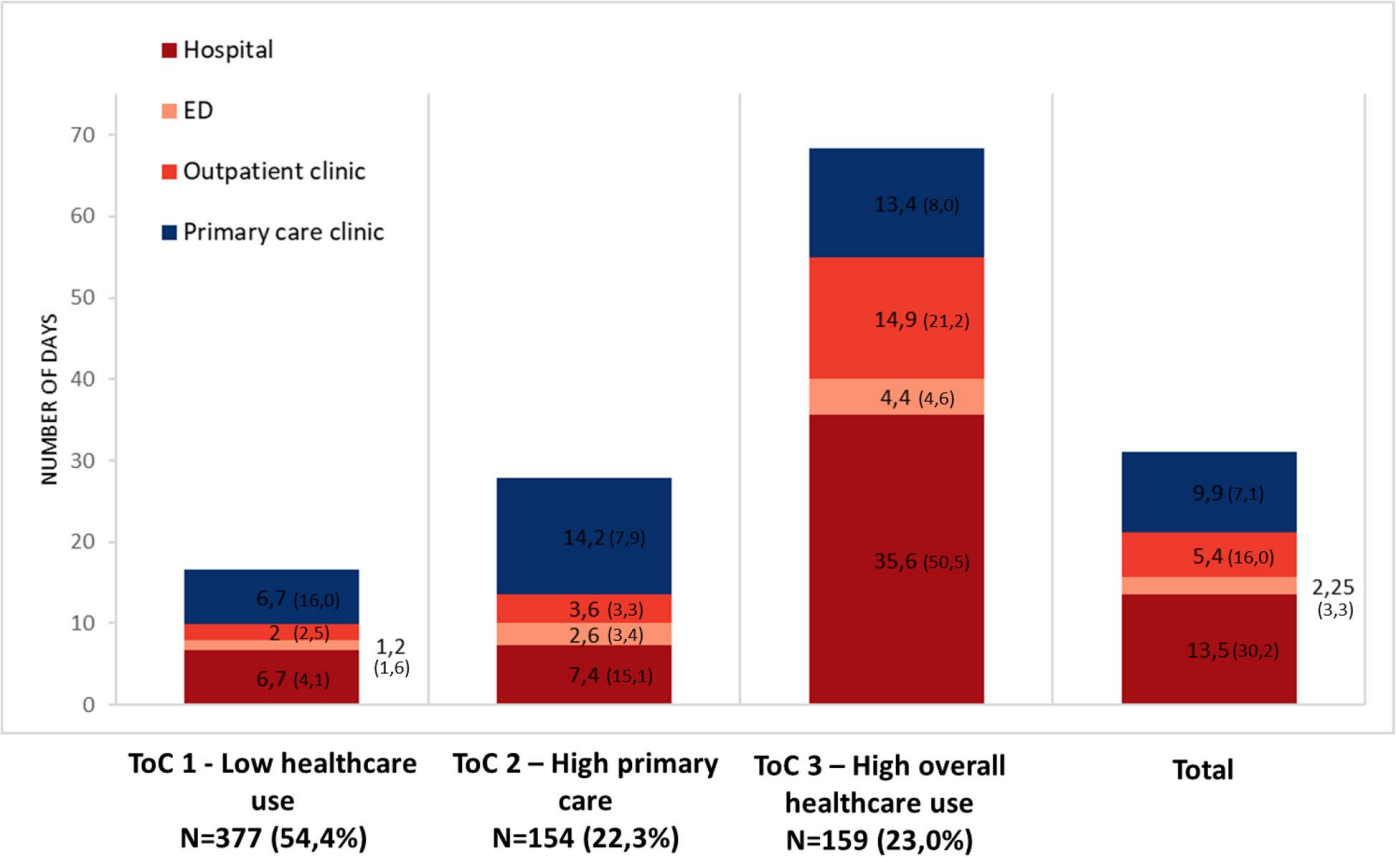
Mean number of days (SD) spent in each state for the where dimension, by ToC type. Abbreviations: ED, Emergency department; SD, Standard deviation; ToC, Trajectory of care

Figure 1 presents the chronogram for each time unit point (24 months) for each group and dimension (*where* and *which*), while Fig. 2 illustrates the mean number of days spent in each care setting for the w*here* dimension.

Table 1 presents individual characteristics and shows that those characteristics differ according to group membership. Individuals in group 1 (*low healthcare use*) had the lowest proportion of overall healthcare use, with a mean of 6.7 primary-care visits over two years (SD = 16.0). They are notably characterized by being older (mean = 81.7; SD = 6.6), having a better perceived health status (*n* = 137; 36.4%), and having the lowest comorbidity index (mean = 3.3; SD = 3.0). Individuals in group 2 (*high primary care use*) had the most primary care visits over two years (mean = 14.2; SD = 14.2). This group mainly consisted of females (*n* = 111; 72.1%) and individuals most likely to live in rural areas (*n* = 55; 35.7%). The proportion of all their health conditions and healthcare use lies between groups 1 and 3. Individuals from group 3 (*high overall healthcare use*) reported the highest healthcare use, with a higher mean number of days spent in hospitals (mean = 35.6; SD = 50.5), EDs (mean = 4.4; SD = 4.6), and outpatient clinics (mean = 14.9; SD = 31.2) during the two-year period. Individuals in this group were less likely to be female (*n* = 77; 48.4%), as well as being more likely to live in metropolitan areas (*n* = 99; 62.3%) and to report a poor/fair perceived health status (*n* = 94; 59.2%). They also presented with a higher comorbidity index (mean = 7.1; SD = 4.4), mean number of monthly medications (mean = 21.4; SD = 8.6), reported more common (*n* = 61; 38.4%) and severe (*n* = 36; 22.6%) mental health disorders, and experienced a higher death rate in the two years after the index date (*n* = 33; 20.8%).

**Table 1:** Characteristics of individuals, according to their respective ToC type

Variables	All individuals *N* = 690	1—Low healthcare use *n* = 377 (54.6%)	2- High primary care use *n *= 154 (22.3%)	3- High overall healthcare use *n* = 159 (23.0%)	*p*-value^a^
**Socioeconomic**					
Age^b^	80.8 ± 6.7	81.7 ± 6.6	80.6 ± 6.6	79.2 ± 6.8	0.0004
Sex (female)	435 (63.0)	247 (65.5)	111 (72.1)	77 (48.4)	< .0001
Living alone	309 (44.8)	166 (44.0)	72 (46.8)	71 (44.7)	0.8484
Individual annual income more then 20,000$	203 (39.3)	174 (63.3)	80 (52.0)	60 (48.8)	0.0060
Education level Post-secondary or higher	265 (40.2)	138 (38.4)	51 (34.7)	76 (49.4)	0.0215
Residential area					
Metropolitan	353 (51.2)	181 (48.0)	73 (47.4)	99 (62.3)	0.0177
Small town	142 (20.6)	90 (23.9)	26 (16.9)	26 (16.9)	
Rural	195 (28.3)	106 (28.1)	55 (35.7)	34 (21.4)	
Perceived health status (Poor/Fair)	294 (42.7)	137 (36.4)	63 (40.9)	94 (59.1)	< .0001
**Health conditions**					
Months since dementia identification^b^	36.9 ± 35.2	39.3 ± 34.8	35.6 ± 38.2	32.3 ± 32.6	0.1001
Dementia identified during the index period (yes)	182 (26.4)	90 (23.8)	45 (29.2)	47 (29.56)	0.1203
Comorbidity index^b^	3.7 ± 3.4	3.3 ± 3.0	4.4 ± 3.2	7.1 ± 4.4	< .0001
Comorbidity index					
0–3	353 (51.2)	246 (65.3)	72 (46.8)	35 (22.0)	< .0001
4 and over	337 (48.8)	131 (34.8)	82 (53.3)	124 (78.0)	
High blood pressure	304 (44.1)	120 (31.8)	78 (50.7)	106 (66.7)	< .0001
COPD	120 (17.4)	45 (12.0)	29 (18.8)	46 (28.9)	< .0001
Diabetes	138 (20.0)	58 (15.4)	31 (20.1)	49 (30.8)	0.0002
Heart disease	225 (32.61)	74 (19.6)	54 (35.1)	97 (61.0)	< .0001
Cancer	40 (5.8)	Less than 15	Less than 15	25 (15.7)	< .0001
Common mental-health disorder	155 (22.5)	50 (13.3)	44 (28.6)	61 (38.4)	< .0001
Severe mental-health disorder	76 (11.0)	21 (5.6)	19 (12.3)	36 (22.6)	< .0001
**Healthcare use**					
Regular primary care provider	508 (95.9)	269 (94.4)	116 (95.9)	123 (99.2)	0.0813
Primary care provider continuity of care^b^	0.90 ± 0.18	0.914 ± 0.164	0.89 ± 0.19	0.869 ± 0.201	0.0309
Death within 2 years after index date	80 (11.6)	34 (9.0)	Less than 15	33 (20.8)	0.0002
Long-term care admission within 2 years after the index date	92 (13.33)	53 (14.06)	20 (12.99)	19 (11.95)	0.7982
**Medication**					
Average number of monthly medications^b^	12.1 ± 6.7	11.8 ± 5.8	16.0 ± 7.46	21.4 ± 8.6	< .0001
Benzodiazepine	276 (42.1)	127 (35.8)	70 (46.7)	79 (52.7)	0.0009
Anticholinergic	478 (73.0)	231 (65.1)	111 (74.0)	136 (90.7)	< .0001
Antipsychotic	145 (22.1)	7 (21.7)	31 (20.7)	37 (24.7)	0.6750
Opioid	115 (17.6)	38 (10.7)	28 (18.7)	49 (32.7)	< .0001

*Abbreviations*: *COPD* chronic obstructive pulmonary disease, *SD* standard deviation

^a^ Differences between ToC type were tested using ANOVA (continuous variables) and Chi-square test (categorical variables)

^b^ Continuous variable reported using mean ± SD, all other variables are reported using n(%)

**Fig. 3:**
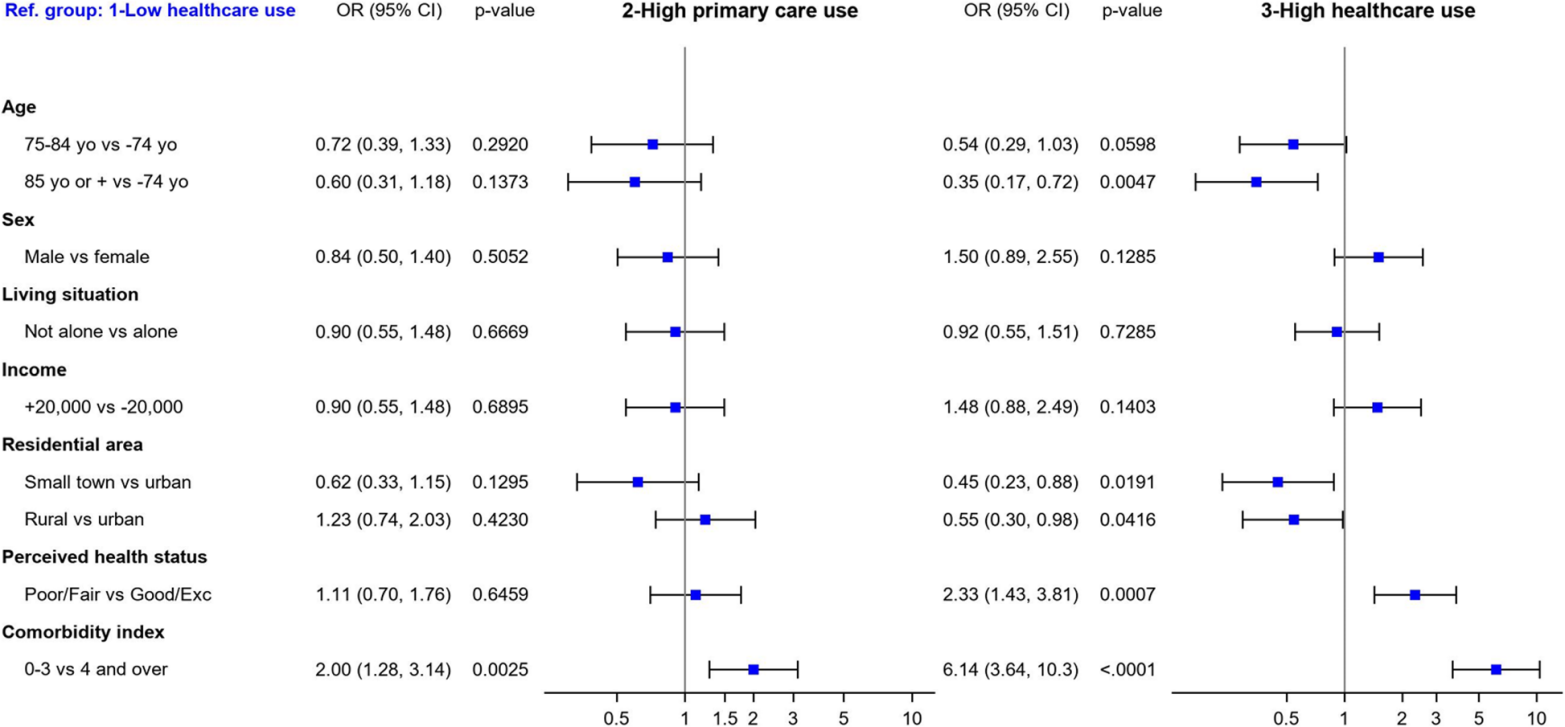
Association between group membership, socioeconomic factors, and self-perceived health. Abbreviations: OR, Odd ratio; CI, Confidence interval; Yo, Years old; Exc, Excellent

Figure 3 presents the multinomial logistic regression results aiming to evaluate the association between ToC type membership, socioeconomic factors, and self-perceived health while accounting for comorbidity (with group 1 being the reference group). Group 3 membership was associated with younger age, living in urban areas, a poorer perceived health status, and a higher comorbidity index. A higher comorbidity index was the only variable significantly associated with group 2 membership.

## Discussion

We aimed to identify the ToCs of people living with dementia in the community and to evaluate the association of socioeconomic factors and self-perceived health with ToC membership. Three groups emerged: (1) *low healthcare use; (2) high primary care use; and (3) high overall healthcare use.* The results from our regression model show that lower perceived health and specific socioeconomic factors (younger age, living in urban areas) were associated with *high overall healthcare use* (relatively to *low healthcare use*). A higher comorbidity index was also associated with membership in groups 2 and 3.

These results contribute to an emerging literature on ToCs for persons living with dementia [12]. Nevertheless, comparing our results with existing studies is challenging. Indeed, their approach often encompasses a global population or is oriented toward other services such as long-term care placement or end-of-life years [14, 24, 25].

Our results suggest a significant variation in healthcare use within our population and are supported by scientific literature. First, the characteristics of group 1 (*low healthcare use*) could indicate that most individuals could manage their health conditions with minimal healthcare services and visits to general practitioners. Indeed, this group had few primary care visits to these professionals, while the yearly rate for the overall population with dementia could be around 7 to 10 visits to general practitioners [26]. Their lower comorbidity rates could explain this limited healthcare use, as higher comorbidity is associated with more healthcare use and costs [27, 28]. The patterns of healthcare use in this group allow us to hypothesize that this group gathers the less severe dementia cases, as functional and cognitive deficits are also associated with higher rates of healthcare use, including hospitalizations [27, 29].

In parallel, group 2 (*high primary care use)* comprised individuals with higher comorbidity and the highest number of visits to primary care clinics. Studies have shown that those living with dementia present higher patterns of primary care use. This is especially true around critical periods when healthcare needs could be more significant, such as diagnosis and end-of-life years [28, 30]. Thus, the rate of consultations with cognition specialists in this group may indicate higher severity of dementia (lying between groups 1 and 3), mainly managed in primary care settings. In Canada, guidelines also recommend that the treatment and care of patients with dementia be placed under the responsibility of family medicine groups (FMGs), with the collaboration of secondary and tertiary care for complex cases [31].

Group 3 (*high overall healthcare use*) comprises individuals with the highest comorbidity and overall use of healthcare services. Individuals with dementia and high comorbidity tend to have higher polypharmacy and healthcare use [32]. This group's higher rates of severe mental health conditions could further complicate dementia management. Indeed, persons living with dementia and presenting a psychiatric comorbidity are more likely to use medical inpatient services than those without psychiatric comorbidities [33].

Our second objective was to evaluate the association between ToC membership, socioeconomic factors, and self-perceived health while accounting for comorbidity. Mainly, the result shows that living in urban areas, lower perceived health status is associated with group 3 membership, while higher comorbidity is associated with membership to groups 2 and 3. This last result is not surprising, as comorbidity is associated with higher healthcare use in the population with and without dementia [34].

Regarding socioeconomic factors, studies showed that individuals living with dementia in urban areas have more ED visits and hospital use than their rural and micropolitan counterparts. This might be related to service availability and a regional propensity to seek such types of care [13, 35]. These patterns of healthcare use, however, put the individuals at higher risk of adverse effects, notably as longer lengths of stay in EDs and hospital environments predispose patients living with dementia to more hospital harm (including delirium and higher mortality) [35]. Lastly, membership in group 3 was associated with lower self-perceived health. Self-rated health is widely used for assessing health in older adults. It is negatively influenced by comorbidity, polypharmacy, poor mental health, functional outcomes, and healthcare use [36, 37], which is consistent with our results.

### Implications for practice and research

Persons living with dementia present with a range of medical and cognitive needs, underscoring that models of care ought to incorporate this medical complexity to improve the management of the condition [38].

For example, the high healthcare use characterizing individuals from group 3 shows they could benefit from intensive interventions to ensure coordination and communication between the various services they already use. Innovative approaches to care delivery, including a dedicated navigator to support the patient throughout the care trajectory, could have a positive impact [39, 40], as the number of transitions tends to decrease with better use of services and support [41].

Polypharmacy was present in all our groups, including group 1, with less healthcare use and low comorbidity. This calls for educating healthcare professionals on medication management, such as with the help of initiatives such as *Choosing Wisely,* which advocates for avoiding unnecessary medical treatments [42]. For complex individuals with a higher level of polypharmacy, such as in group 3, an interdisciplinary approach engaging pharmacists could also be considered. In primary care teams, medication reviews led by a pharmacist can minimize the adverse effects of polypharmacy and improve medication management, including in vulnerable geriatric populations [43, 44].

The use of a ToC approach is also congruent with a care pathway management approach, which describes the optimal course and service to be provided to the right person, in the proper sequence, at the right time, in the right place, and with the best results while focusing on the user's experience [45]. The research on ToCs is of significant value to care planning and organization. It illuminates how real-life ToCs differ from the ideal ones and how they vary concerning inequities [46]. For example, care trajectories can help contextualize the timing and nature of different care transitions and identify the types of services an individual may require based on their characteristics.

However, formal healthcare use may not reflect actual healthcare needs of people living with dementia. To fully understand an individual’s care trajectory, one must consider their use of other healthcare services, such as home care and informal care. In any three ToC, individuals may present unmet needs in many spheres, including safety, daily activities, and managing cognitive symptoms [47]. Considering the trajectory of cognitive decline is also crucial for services planning, as it may significantly impact healthcare use and costs throughout the disease [48].

For future research, the development of ToCs should be disaggregated within specific geographic areas to inform service planning for individuals living with dementia. Our groups could also be further described and understood regarding key aspects (including the severity of dementia, frailty index, intensity, and type of home-care services used) and moments (i.e., end-of-life years, time of diagnosis). Future research incorporating an overall measure of deprivation could also give insight into how ToCs and needs vary within specific geographical areas.

### Strength and limitations

First, our study uses an innovative cohort in Quebec (the TorSaDe cohort), which links survey and health administrative data. We identified ToCs using a robust SSA approach, which allows the development of homogeneous ToC types to better capture patterns of healthcare use within a complex population. Our multidimensional approach allowed the inclusion of nine states, partitioned into two distinct sequence dimensions, facilitating interpretation and visualization [49, 50].

However, data limitations prevented us from considering important variables related to patient clinical attributes (e.g., dementia severity). Also, our analysis was limited by the exclusion of nursing and community-based services. Specifically, individuals using lower levels of formal healthcare services, such as those in ToC 1, may still present a high level of healthcare use that was not captured in our analysis.

Furthermore, the choice of our index period (two years before the index date of the CCHS) limited the inclusion of people with more severe dementia (as about 26.4% were diagnosed during the index period), in end-of-life situations or near death and might have introduced a bias in favor of healthier individuals (possibly compounding the bias inherent with considering community-dwelling persons with dementia, as access to long-term care facilities in Quebec is scarce and limited to the most severe cases). This choice was consecutive to sample size and the respect of data confidentiality standards. Lastly, we could not consider immigration status in our regression model for confidentiality purposes related to low-frequency cell counts. To overcome these limitations, an updated database could provide access to a more extensive sample. A mixed methods approach could also be employed to consider patients' clinical attributes. This approach would involve combining qualitative and quantitative methods to capture a more comprehensive understanding of the complex interactions between patient characteristics and healthcare utilization.

## Conclusions

This is the first study to propose a comprehension assessment of shared patterns of healthcare use among persons living with dementia in the community, which is a complex and heterogeneous population. Understanding how subgroups of patients use healthcare services over time helps highlight areas of fragility in allocating geriatric-care resources to implement best practices and in the context of resource shortage.

## Supplementary Information

**Additional file 1.** International Classification of Diseases (ICD) codes used for the measures of chronic conditions.

**Additional file 2. **Past and current members of theTORSADE Cohort Working Group. 

## Data Availability

The data from the TorSaDE cohort are not publicly available and are under strict security and confidentiality regulations. Please contact the corresponding author for more information.

## Abbreviations

CCHS: Canadian Community Health Survey
DHD: Dynamic hamming distance
ED: Emergency department
ICD: International Classification of Diseases
RAMQ: Régie de l’assurance maladie du Québec
SSA: State sequence analysis
TorSaDE: Care Trajectories—Enriched Data
ToCs: Trajectories of care
