# Supplementary material for: Trajectories of care of community-dwelling people living with dementia: a multidimensional state sequence analysis
Source: BMC Geriatr. 2023 Apr 27;23:250. doi: 10.1186/s12877-023-03926-x (PMC10134621; doi:10.1186/s12877-023-03926-x)
Supplement: Supplementary file 1 — Additional file 1. International Classification of Diseases (ICD) codes used for the measures of chronic conditions. [file 12877_2023_3926_MOESM1_ESM.docx]

**Additional file 1.** International Classification of Diseases (ICD) codes used for the measures of chronic conditions

| **Conditions** | **IC9-CM** | **IC10-CA** |
| --- | --- | --- |
| **High blood pressure** | 401/ 402 /403/ 404/ 405/ 4372 | I10/ I11/ I12/ I13/ I15/ I674 |
| **Chronic obstructive pulmonary disease** | 490/ 491/ 492/ 493/ 494/ 495/ 496/ 497/ 498/ 499/ 500/ 501/ 502/ 503/ 504/ 505/ 5064/ 5081 | J40/ J41/ J42/ J43/ J44/ J45/ J46/ J47/ J60/ J61/ J62/ J63/ J64/ J65/ J66/ J67/ J684/ J701/ J703 |
| **Diabetes** | 2500/ 2501/ 2502/ 2503/ 2504/ 505/  2506/ 2507/ 2508/ 2509 | E100/ E101/ E109/ E110/ E111/ E119/ E130/ E131/ E139/ E140/ E141/ E149/ E102/ E103/ E104/ E105/ E106/ E107/ E108/ E112/ E113/ E114/ E115/ E116/ E117/ E118/ E132/ E133/ E134/ E135/ E136/ E137/ E138/ E142/ E143/ E144/ E145/ E146/ E147/ E148 |
| **Heart disease** | 4260/ 4267/ 4269/ 4270/ 4271/ 272/ 4273/ 4274/ 4276/ 4277/ 4278/ 279/ 7850/ V450/ 533/ 428/ 4021/ 4041/ 430/ 431/ 432/ 433/ 434/ 435/ 436/ 437/ 438/ 394/ 395/ 396/ 397/ 424/ 7463/ 7464/ 7465/ 7466/ 422/  V433/ 410/ 412/ | I47/ I48/ I49/ I441/ I442/ I443/ I456/ I459/ R000/ R001/ R008/ T821/ Z450/ Z950/ I50/ G45/ G46/ I60/ I61/ I62/ I63/ I64/ I65/ I66/ I67/ I68/ I69/ I05/ I06/ I07/ I08/ I34/ I35/ I36/ I37/ I38/ I39/ I091/ I098/ Q230/ Q231/ Q232/ Q233/ Q238/ Q239/ Z952/ Z953/ Z954/  I21/ I22/ I252/ |
| **Cancer** | 14/ 15/ 16/ 17/ 18/ 19/ 20/ 21/ 22/ 23 | C0/ C1/ C2/ C3/ C4/ C5/ C6/ C7/ C8/ C9/ D0/ D1/ D2/ D3/ D4 |
| **Common mental health disorders** | 300/ 311/ 309/ 2961/ 2963/ 3004/ 3102/ 3119/ | F32/ F33/ F40/ F41/ F42/ F44/ F48/ F204/ F313/ F314/ F315/ F341/ 412'/ 'F432'/ 'F530'/ 'F432 |
| **Severe mental health disorders** | 295/ 296/ 297/ 298/ 2980 | F20/ F21/ F22/ F23/ F24/ F25/ F28/  F29/ F30/ F31/ F232 |
