# Supplementary material for: Trajectories of care of community-dwelling people living with dementia: a multidimensional state sequence analysis
Source: BMC Geriatr. 2023 Apr 27;23:250. doi: 10.1186/s12877-023-03926-x (PMC10134621; doi:10.1186/s12877-023-03926-x)
Supplement: Supplementary file 2 — Additional file 2. Past and current members of theTORSADE Cohort Working Group. [file 12877_2023_3926_MOESM2_ESM.docx]

**Additional file 2.** Past and current members of the TORSADE Cohort Working Group

Alain Vanasse (outgoing director), Alexandre Lebel, Amélie Quesnel-Vallée, Anaïs Lacasse (incoming director), André Néron, Anne-Marie Cloutier, Annie Giguère, Benoit Lamarche, Bilkis Vissandjee, Catherine Hudon, Danielle St-Laurent, David Buckeridge, Denis Roy, Geneviève Landry, Gillian Bartlett, Guillaume Blanchet, Hermine Lore Nguena Nguefack, Isabelle Leroux, Jaime Borja, Jean-François Ethier, Josiane Courteau, Lucie Blais, Manon Choinière, Marc Dorais, Marc-André Blanchette, Marc-Antoine Côté-Marcil, Marie-Josée Fleury, Marie-Pascale Pomey, Mike Benigeri, Mireille Courteau, Nadia Sourial, Pasquale Roberge, Pier Tremblay, Pierre Cambon, Roxanne Dault, Sonia Jean, Sonia Quirion, Stéphanie Plante, Thomas Poder, Valérie Émond.
